# Electrostatic landscapes in crystal engineering: a new perspective on synthons

**DOI:** 10.1107/S2052252525003148

**Published:** 2025-04-14

**Authors:** Alexander S. Novikov

**Affiliations:** a Institute of Chemistry Saint Petersburg State University Universitetskaya Nab. 7/9 199034Saint Petersburg Russian Federation; b Research Institute of Chemistry, Peoples’ Friendship University of Russia (RUDN University), Miklukho-Maklaya Str. 6, 117198Moscow, Russian Federation

**Keywords:** crystal engineering, synthons, electrostatic interactions, charge density analysis

## Abstract

The design of crystalline solids relies on understanding and controlling intermolecular and intramolecular interactions. Through theoretical charge density analysis and database mining, Shukla *et al.* [(2025). *IUCrJ*, **12**, 334–357] have found a new way of viewing supramolecular assembly through the lens of electrostatic complementarity.

Crystal engineering, the art and science of designing crystalline solids with desired properties, relies heavily on understanding and controlling intermolecular and intramolecular interactions (Desiraju, 2013 ▸). For decades, the focus has been on identifying and utilizing ‘synthons’ (Desiraju, 1995 ▸) – recurring structural units formed through non-covalent interactions like hydrogen, halogen and chalcogen bonds (Dhaka *et al.*, 2024 ▸; Dukhnovsky *et al.*, 2024 ▸). Now, in this issue of *IUCrJ*, Shukla *et al.* (2025 ▸) push the boundaries of this field by demonstrating that the origin of these synthons lies not in the specific atoms or functional groups involved, but rather in the underlying electrostatic complementarity between electrophilic and nucleophilic regions.

The authors use a combination of theoretical charge density analysis (density functional theory calculations together with topological analysis of the electron density distribution within the QTAIM framework) and Cambridge Structural Database (CSD) mining to dissect the nature of halogen, chalcogen and hydrogen bonds, and answer the question: ‘Is the origin of the motif associated with the atom or the functional group involved in the formation of the interactions building the supramolecular structure, or with the appropriate orientation of particular electrophilic and nucleophilic regions present on the interacting atoms?’. Their central argument revolves around the idea that these interactions, regardless of the atoms involved, are fundamentally driven by the attraction between regions of charge depletion (electrophilic) and charge concentration (nucleophilic) on the interacting molecules.

The study begins with an intriguing observation: the crystal structure of 4-iodo-1,3-di­thiol-2-one (IDT) features a four-membered ring motif formed by S⋯S and S⋯I chalcogen bonds (Fig. 1 ▸). Remarkably, this motif closely resembles one found in seleno­phthalic anhydride (SePA), which is stabilized by Se⋯Se and Se⋯O chalcogen bonds. This structural similarity, despite the different atoms involved, prompts the key question: what dictates the formation of these recurrent supramolecular motifs?

Through rigorous charge density analysis, Shukla *et al.* reveal that the chalcogen bonding interactions in IDT and SePA exhibit the same characteristic orientation of local electrostatic electrophilic⋯nucleophilic interactions. This suggests that the specific atoms are less important than the spatial arrangement of charge-depleted and charge-concentrated regions.

To generalize their findings, the authors delve into the Cambridge Structural Database (CSD), a vast repository of crystal structures. By analyzing synthons and supramolecular motifs formed by chalcogen-, halogen- and hydrogen-bonding interactions, they demonstrate a consistent pattern: geometrical preferences in molecular assembly are governed by the relative positions of charge concentration (CC) and charge depletion (CD) sites. These sites tend to align along internuclear directions, even with relatively weak interaction energies. Interestingly, the authors have proposed several geometrical descriptors based on intermolecular contact angles, intramolecular angles and planarity degree angle as a good tool to discern among different chalcogen⋯chalcogen contacts, and in particular to assess δ^+^⋯δ^−^ interactions involving chalcogen atoms.

Furthermore, the authors propose a refinement of the graph-set notation commonly used to describe hydrogen-bonded networks. They suggest replacing the traditional ‘donor–acceptor’ terminology with ‘nucleophilic–electrophilic’ to better reflect the underlying electrostatic nature of these interactions. This subtle but significant change emphasizes the importance of charge complementarity in driving supramolecular assembly.

I believe that the work of Shukla *et al.* has significant implications for crystal engineering. By shifting the focus from specific functional groups to the broader concept of electrostatic complementarity, researchers can gain a more fundamental understanding of supramolecular interactions. This, in turn, could lead to more rational design strategies for co-crystals, organic materials and other functional solids. The ability to predict and control the formation of specific supramolecular motifs is crucial for tailoring the properties of crystalline materials. Shukla *et al.*’s findings suggest that by carefully considering the electrophilic and nucleophilic properties of molecular building blocks, it may be possible to design materials with predictable and desirable structures.

In conclusion, this study provides a compelling case for viewing supramolecular assembly through the lens of electrostatic complementarity. By moving ‘beyond atoms and functional groups’, Shukla *et al.* offer a new perspective on the fundamental forces that govern the organization of molecules in the solid state. This work not only deepens our understanding of non-covalent interactions but also paves the way for more sophisticated and rational approaches to crystal engineering.

I also believe that future studies of the influence of different charge decomposition schemes (*e.g.*, Hirshfeld atomic charges, Voronoi deformation density atom population, Mulliken/Löwdin/NBO population analysis, Becke atomic charges with atomic dipole moment correction, electrostatic surface potential fitting atomic charges, AIM atomic charges) on the prediction of the electrophilic and nucleophilic properties of molecular building blocks, as well as results of energy decomposition analysis based on dispersion-corrected density functional theory (Lu & Chen, 2023 ▸), could also be helpful for understanding the nature and driving forces of supramolecular assembly formation, and researchers will focus their attention on these points in future work.

## Figures and Tables

**Figure 1:**
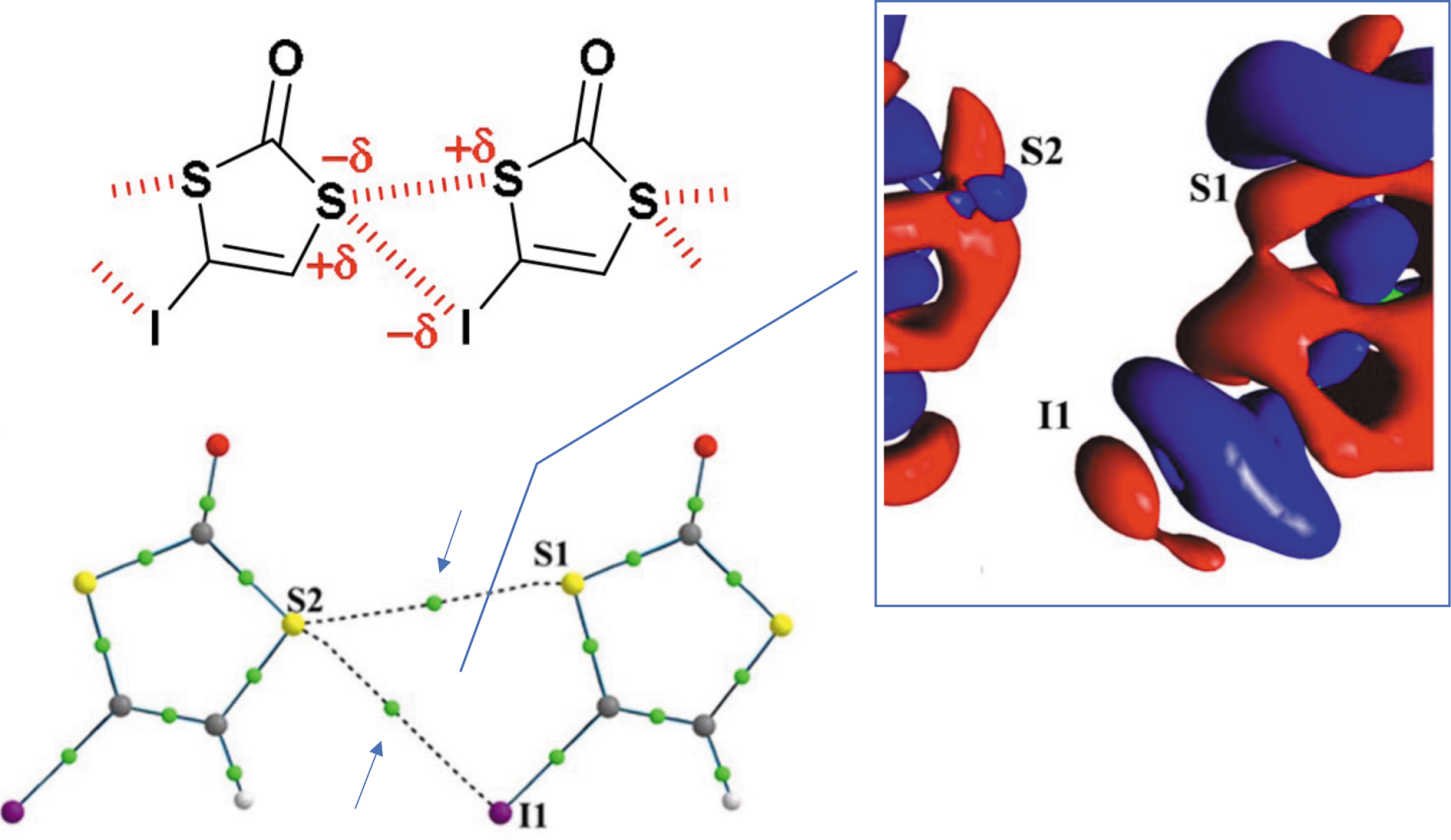
An example of a supramolecular motif investigated by Shukla *et al.* (2025) ▸. Intermolecular contacts are shown with the bond paths and bond critical points between the interacting atoms highlighted by arrows. The static deformation density map in the intermolecular region (positive δ^+^ and negative δ^−^ isosurfaces are in blue and red, respectively) is also shown. [Adapted from Shukla *et al.* (2025) ▸.]
